# Does Equine Interaction Facilitate Emotional Safety and Learning for College Students within an Agricultural-Based Classroom?

**DOI:** 10.3390/ejihpe13110172

**Published:** 2023-11-02

**Authors:** Katie Holtcamp, Molly C. Nicodemus, Tommy Phillips, David Christiansen, Brian J. Rude, Peter L. Ryan, Karen Galarneau

**Affiliations:** 1 Counseling Services, Dogwood Wellness Group, P.O. Box 1016, Starkville, MS 39760, USA; 2 Animal & Dairy Sciences Department, Mississippi State University, Box 9815, Starkville, MS 39762, USA; 3 School of Human Sciences, Mississippi State University, 255 Tracy Drive, Starkville, MS 39762, USA; 4 Large Animal Medicine Department, College of Veterinary Medicine, Mississippi State University, P.O. Box 6100, Starkville, MS 39762, USA; 5 Office of Provost and Executive Vice President, Mississippi State University, P.O. Box BQ, Starkville, MS 39762, USA

**Keywords:** equine interaction, emotional safety, memory development, agricultural-based education

## Abstract

Effective teaching requires an educational environment that promotes learning, and yet, developing such an environment can be challenging within today’s agricultural-based classroom for educators due to the trend to a more virtual teaching format and less hands-on learning. Animal interaction, particularly equine activities, has been shown to assist educators in the development of an emotionally safe environment for promoting learning. However, research is lacking as to whether the interaction with the animal needs to be direct or indirect within the collegiate educational environment to observe benefits. Therefore, the objective of this study was to determine the impact of equine interaction, both direct and indirect, within an educational environment on the emotional safety and learning for the college student within the agricultural-based classroom. Three course types were observed within the agricultural-based educational environment that included courses with no equine interaction (Group A) and courses with equine interaction, both direct (Group B) and indirect (Group C) interaction with the horse. Indirect interaction included items such as observation of equine handling via a video or gaining knowledge from reading online materials, but not engaging in direct, hands-on activities with the horse. Development of emotional safety within the students enrolled within these courses was measured using a self-reporting emotional safety evaluation. Due to the structure of the scale, a decrease in emotional safety indicated a positive change. Learning, both development of semantic and procedural memory, was measured using a student-completed knowledge examination and an instructor-completed skill evaluation, respectively. While significant improvement in emotional safety was not observed within any of the course types, a weak negative correlation was found between emotional safety and semantic memory for students enrolled in equine courses, both direct (R = −0.55, R^2^ = 0.28) and indirect (R = −0.25, R^2^ = 0.06) interaction, finding as emotional safety scores lowered to the ideal range that knowledge improved. In addition, students within equine courses showed semantic memory development in specific areas of equine sciences (Group B: Grooming/Tacking, *p* = 0.03; Group C: Equine Behavior, *p* = 0.04) and direct equine interaction resulted in development of equine-based procedural memory in all four skill areas measured within the study (*p* = 0.00). As such, learning is promoted through equine interaction, whether direct or indirect interaction, within the agricultural-based classroom, suggesting that both forms of equine interaction can be a valuable educational tool for the instructor within the collegiate setting.

## 1. Introduction

Mental health challenges are present on college campuses within the student population, and these challenges can be devastating for the learning potential of students struggling with these issues if not given the right learning environment for success. Mental health diagnoses prior to the COVID-19 pandemic were reported in 35% of first-year college students surveyed according to Eva [1]. This statistic unfortunately did not improve after 2020 as students faced “the greatest disruption in educational opportunity in a generation” with students experiencing “negative effects on physical and mental health” with the loss of the school environment and associated isolation [2]. While educators worked to accommodate students as the collegiate setting moved more aggressively to an online formatting, many educators in academia were limited in their background concerning effective teaching methods for virtual learning [3]. Furthermore, effective teaching became more challenging when addressing unique classroom expectations such as laboratory-based classes associated with agricultural-based programs [4]. The challenge of converting agricultural-based coursework to an online learning platform while maintaining effective instructional delivery has been daunting for students as well [5]. While most colleges today have the option of online or in-person classroom environments, the trend towards a more prevalent online classroom formatting continues within academia even as we move into the post-COVID era [6].

### 1.1. Addressing Classroom Challenges

Agricultural-based educators have a unique tool in assisting students within the in-person classroom environment, which is the presence of an animal in laboratory-based courses. Educational presentations utilizing live animals have shown benefits in engaging students in the learning process [7]. The horse in particular has been shown to promote an emotionally safe environment for promoting learning in adolescents [8]. In fact, equine interaction is a form of experiential learning, which is an emerging topic of conversation in the mental health field. Many treatment facilities across the nation are utilizing experiential approaches to complement their classroom or group-based therapeutic interventions [9]. Experiential learning theory (ELT) is based on the premise that knowledge is gained through participation in physical experiences [10]. Due to the physiological interaction between horses and humans when they share an environment [11], equine-assisted services are the perfect example of ELT in action when conducted through the lens of emotional safety.

Student emotional safety and learning potential are negatively impacted when mental health challenges go untreated [12]. According to the National Center on Safe Supportive Learning Environments [13], an emotionally safe learning environment is one where the student feels “valued, respected, and connected to and engaged in learning” so that the student within the learning environment can “recognize and manage emotions.” This type of learning environment encourages the student to be confident to face new challenges and try something new during their learning process. Creating an emotionally safe learning environment within the college classroom, nevertheless, became challenged with the COVID-19 pandemic as college students moved from in-person to online learning [6]. Students were reported to feel disconnected from the learning process and a prevalence in inequality within education was observed as some students were left behind creating a sense of isolation [14]. This isolation can result in potential unhealthy behaviors as seen in a survey by the American College Health Association [15] in which 20.9% of students reported illicit substance abuse and 24.1% participated in binge drinking during the beginning months of the pandemic, from March through May of 2020.

To combat this sense of isolation and support the mental health of the student, a relationship between the student and educator must be established [4]. This relationship, however, can be difficult to build with a student struggling with mental health challenges [5]. Animals have been used as a therapeutic tool for individuals battling mental health conditions, finding individuals are more willing to participate within the therapeutic intervention in the presence of the animal [16,17,18]. Animal interaction is an effective way to instill and expand empathy within the youth population and foster an environment of unconditional acceptance, encouraging the active participation in the learning process [19]. In addition, according to Ewing et al. [19], the use of the horse has been promising, finding the unique environment of this animal is effective in breaking down barriers for open communication that promotes engagement. Within the educational environment, Silva dos Santos et al. [7] reported the outdoor setting associated with animal-based educational programs assisted in engaging the program participants. The authors further discuss the benefits of live animal educational presentations helping to increase interest in the material discussed, and in turn, promoting the learning process.

While the COVID-19 pandemic necessitated the use of e-learning within the collegiate setting, this move of the classroom online aligned with the trend in learning approaches observed within the current student population, with as much as 90.6%, according to Muca et al. [20], reporting the use of portable electronic devices for educational purposes and activities. Although Muca et al. [20] reported limited digital resource usage for veterinary students, the authors suggest for educators the development of digital educational tools to facilitate engagement and digital readiness of their students. Nonetheless, the transition to e-learning utilizing these digital educational tools restricts the ability of agricultural educators to utilize in-person live animal demonstrations and presentations. This new learning space, however, may not allow for direct interaction with the animal, but indirect interaction is still possible through online presentations. According to Silva dos Santos et al. [7], even indirect interaction with animals kept participants engaged in learning. Wulff-Risner and Stewart [21] reported that structure associated with classroom video presentation compared to live animals was more beneficial to learning for youth participating in a 4-H horse judging program. The authors further discussed the benefit of video equine evaluation activities over in-person horse judging due to the lack of distractions from the live animal that can hinder the learning of procedural memory.

The learning environment, however, needs to foster emotional safety within the students to accomplish effective teaching [12]. Muca et al. [22] goes on to emphasize the importance of the student’s well-being suggesting educators should establish a learning environment that promotes a sense of well-being so that academic performance can be enhanced and further mental health challenges can be minimized. Through equine interaction, Cagle-Holtcamp et al. [8] reported that an emotionally safe environment was achieved that promoted development of both semantic and procedural memory within at-risk youth participating in a six-week horsemanship program. The emotional reaction to the animal within the learning environment assisted with memory development and this was also reported by Silva dos Santos et al. [7] utilizing a wildlife interaction program. Similarly, Polheber and Matchock [23] reported that undergraduate college students tasked with having to prepare a short presentation demonstrated reduced anxiety both physiologically and behaviorally after participating in human–animal interactive activities. These reductions have been linked to the heart-coupling effect, particularly between horses and humans, which has resulted in lowering of physiological stress parameters in humans [23,24]. Baldwin et al. [25] observed, through mindful grooming activities during equine-assisted learning, that older adults demonstrated a matching of heart rate variability frequencies with their horses, suggesting a bonding effect emotionally with their equine partner. As for the specific physiological benefits for the college-aged young adult, while research is limited within this age group, Rothkopf and Schworm [26] concluded after the completion of a survey study that human–animal interaction within the collegiate setting holds promise and should be explored by higher education administrators as a way to promote emotional well-being for the academic success of the student.

Although animal interaction has been reported to not only reduce anxiety but also promote semantic and procedural memory [27], Janssens et al. [28] found that simply the presence of the animal, even without the direct interaction, had a positive impact on emotional well-being. Wulff-Risner and Stewart [21] further reported improved semantic memory development with the use of indirect animal interaction. In fact, autistic children were documented to learn proper socialization skills through the use of interactive virtual online pets [29]. Furthermore, Mueller et al. [30] determined that the presence of an animal without direct interaction had the same response as a live animal and went on to conclude that a stuffed animal could have a positive response for adolescents experiencing social anxiety. This sentiment was further supported by Koh et al. [31], finding robotic pets offered potential for improving engagement for individuals struggling with cognitive limitations [31].

### 1.2. Study Purpose

With these studies in mind, the potential of indirect interaction with an animal may be an alternative approach to developing an effective emotionally safe learning environment when direct interaction is not available. Nonetheless, without further research, this conclusion is limited. Therefore, the objective of this study was to determine if equine interaction, directly or indirectly with the horse, within the collegiate educational environment would improve the emotional safety of the student, and in turn, promote learning within an agricultural-based classroom. The hypothesis is that participation in the educational environment centered around equine interaction, whether it included direct or indirect interaction, will result in improved emotional safety and learning, both semantic and procedural memory development, for the college student. This study is of value considering the evolution of today’s college classroom to online formatting and the continued struggles concerning mental health within the college student that hinders the potential for learning. Through this study, we intend to provide data that can inform the agricultural-based educator within the collegiate setting as to the benefit of introducing direct and indirect interaction with the horse within their classroom.

## 2. Materials and Methods

### 2.1. The Participants

College students enrolled at Mississippi State University during the fall 2018 and 2019 semesters were recruited to participate in the evaluation protocol. Student participants for this study were categorized into three groups: Group A—students enrolled in courses without any form of equine interaction; Group B—students enrolled in equine coursework with direct interaction with horses through weekly equine-handling laboratories; and Group C—students enrolled in equine coursework with indirect interaction with horses through weekly course presentations, videos, class assignments, and reading materials. Students were recruited from junior- and senior-level courses. All courses utilized for recruiting for this study were housed under an agricultural-based college program. Nevertheless, the courses were open to all majors and lacked any prerequisites. The courses utilized for this study were all introductory-based courses within the agriculturally-based curriculum. The primary difference between the two courses with equine interaction (Groups B and C) was based mainly on whether the student had direct (Group B) or indirect (Group C) interaction with horses during the coursework. Students could not participate in multiple groups within this study and were sorted into the appropriate category by reviewing class enrollment schedules.

Exclusion criteria for students enrolled in the courses with equine interaction (Groups B and C) were that they could not be enrolled in additional equine-based coursework or participate in additional equine extracurricular activities offered through the University at the time of the study. Similarly, exclusion criteria for students enrolled within the courses without equine interaction (Group A) were that the students could not be enrolled in any type of equine-based coursework or participate in any equine extracurricular activity offered through the University at the time of the study. While survey participation was open to all majors, participants needed to be fulltime undergraduate students enrolled at the University. To ensure consistency between the groups evaluated, all courses utilized for this study were in-person courses, and thus, online or hybrid courses were not included in the current study. According to the above inclusion and exclusion criteria, a total of 142 students were recruited for Group A, 70 students were recruited for Group B, and 71 students were recruited for Group C. Students were recruited through their courses by their course instructors with participation in all aspects of the study being voluntary. No incentives were given for survey participation. No grade nor point value was directed toward survey submission. All aspects of the study protocol were evaluated and approved by the Institutional Review Board at University before the onset of the study.

### 2.2. Evaluation Protocol

The evaluation protocol consisted of a two-part self-reporting survey instrument and an instructor-based skill evaluation. All questions from both the survey instrument and the skill evaluation were reviewed by the research team and professionals from the equine industry before distribution to students to determine effectiveness for reaching study objectives. After a review of questions, the two-part self-reporting survey instrument was made available to all students participating in the study. Due to safety concerns for the students, only those students enrolled in an equine course with weekly direct interaction with the horse in a laboratory setting (Group B) were recruited for the instructor-based skills evaluation. All equine-related activities associated with the skill evaluation were approved by the Institutional Animal Care and Use Committee at University before the onset of the study.

#### 2.2.1. Two-Part Self-Reporting Survey Instrument

The two-part self-reporting survey instrument was distributed both in a paper and online format with both formats utilizing Qualtrics for the recording of data. The online survey instrument was distributed to the students through an email sent out to the students by the instructors of the courses. Email addresses utilized for the survey distribution was those assigned by the University upon the student entering the University and access to email address information was obtained by the course instructors at the time of student course enrollment. Distribution of the survey instrument, both paper and online format, was conducted solely by the instructors for the courses being utilized for the study. Students filling out the paper survey instrument could complete the survey during the class period in which the survey was given to them by their respective instructor or could take the survey home to return the completed survey the following class meeting. The instructor for the courses were responsible for collecting all surveys, and once collected, instructors submitted returned surveys to the research team for analysis. The paper survey instrument was given as an option for students that may have difficulties utilizing the online system. Less than 10% of each of the three groups utilized the paper format, with preliminary analysis finding no significant differences (*p* < 0.05) between the two formats, and thus, both formats were combined for results reporting.

The two parts of the survey instrument consisted of a 60-question emotional safety self-evaluation and a 22-question equine-based knowledge evaluation focused on semantic memory development. Both parts of the survey instrument were completed at the same time with students completing the survey questions independently. Instructors were available for clarification of survey questions and students were given as much time as needed to complete both parts of the survey instrument. Preliminary analysis of the survey determined Cronbach’s alpha for the emotional safety evaluation to be α = 0.95 and for the knowledge evaluation to be α = 0.70, indicating that the survey instrument was reliable.

Emotional Safety Self-Evaluation. All student participants were given a self-reporting emotional safety evaluation at the beginning (pre-survey) and end of the semester (post-survey). The evaluation was a 60-question survey compiled from previously validated evaluations that included GAD-7 [32], Social Connectedness Scale [33], Emotional Needs Scale [34], Self-Esteem Inventor [35], and Trust/Respect Assessment [36]. This combination was selected due to the lack of a previously established measure that could capture the entirety of the newly defined emotional safety components [8,13]. To provide as much validity to the study as possible, the research team chose measures that were already accepted as reliable and put them in one survey for ease of participation. This survey was divided into four categories: Personal Security (Table 1), Respect (Table 2), Self-Esteem (Table 3), and Connectivity (Table 4). When scoring this survey, questions with positive underpinnings were scored as the following: Always—1, Sometimes—2, Seldom—3, Never—4, and N/A—5. Questions with negative connotations were scored reversely as the following: Never—1, Seldom—2, Sometimes—3, Always—4, and N/A—5. In terms of ceteris paribus, or “all other things being equal”, the desired score reflecting a student with a healthy emotional safety would produce 1 point for each of the 60 questions, thus resulting in a score of 60, reflecting the most ideal level of emotional safety. Scoring system followed that given by the previously validated evaluations that were utilized for development of the survey instrument [32,33,34,35,36]. For inclusion within this study, only completely answered surveys were included.

Semantic Memory: Knowledge Evaluation. All student participants were given a pre- and post-equine-based knowledge examination for evaluating development of semantic memory. Topics covered in the examination were items covered within all equine courses participating in this study. The examination was given out at the same time as the emotional safety evaluation with questions being the same for both the pre- and post- evaluations. The questions were adapted from the Certified Horsemanship Association Instructor Manual Level 1 [37]. The exam was broken down into 12 questions consisting of multiple choice (Questions 1–12) and 10 questions in the form of true or false (Questions 13–22), for a total of 22 questions (Table 5). Multiple-choice questions allowed for four possible answers. All questions were forced-choice questions, with no questions allowing for written-in responses. Questions were divided into the following four topic areas in equine sciences: Behavior (Questions 1, 2, 8, and 15); Care (Questions 3 and 11–13); Grooming/Tacking (Questions 4, 10, 14, 16, and 21); and Riding (Questions 5–7, 9, 17–20, and 22). Each correct answer for all 22 questions was given 1 point each, so that a score of 22 would indicate that all questions in the knowledge evaluation were answered correctly. No partial credit was given for any answer.

#### 2.2.2. Procedural Memory: Skill Evaluation

Student participants with direct access to equines through weekly laboratory coursework (Group B) were evaluated for procedural memory development through the instructor-based skill evaluation conducted within the equine environment [38]. The skill evaluation was completed by course instructors during the same week as the self-reporting survey instrument with evaluation questions being the same for both the pre- and post- evaluations. The evaluation consisted of 10 questions that were answered by instructors as they assessed their students’ performance within the laboratory setting. The skills assessed by the instructors within this skill evaluation were activities covered within the laboratory during the course. The course instructor directed the students as to which skills were to be performed during the laboratory period for assessment, and then utilized the evaluation to complete the assessment of the student’s skills that were demonstrated. All evaluations were completed utilizing paper format by the instructor with evaluations submitted to the research team after completion. Students were given unlimited time during the laboratory period to complete the skills requested for the evaluation. The questions were adapted from the Certified Horsemanship Association Instructor Manual-Level 1 [37] (Table 6). Questions were divided into four assessment areas: Abilities in Barn Management (Questions 2–3); Quality in Barn Management (Question 4–5); Abilities in Equine Handling (Questions 1, 6–8); and Skills in Team Building within the Equine Environment (Questions 9–10). A scoring system for the evaluation was established using a 1 to 4 rating scale, with a score of 4 indicating a high level of proficiency and comfort with and around horses. The tasks assessed by the instructor during the laboratory included approaching a horse, management of the equine environment, and basic horse handling with tasks completed both individually and with other students within the course. The instructor marked one of the following for each question: 1—Poor, 2—Needs Improvement, 3—Meets Expectations, or 4—Exceeds Expectations. Scores were summed from the 10 questions for the final score. Instructors were required to score each question for each student. Each participant had the opportunity to score a maximum total of 40 points.

### 2.3. Statistical Analysis

Descriptive statistics (means and standard deviations) were analyzed for each component of the evaluation protocol. A Shapiro–Wilk test for normality was performed and resulted in *p* > 0.05, indicating normal distribution of data. A paired-sample *t*-test was performed using IBM SPSS Statistics 26 (Armonk, NY, USA) to compare the pre- and post- evaluation scores for each component of the evaluation protocol within each group. A one-way ANOVA with post hoc Bonferroni analysis was conducted to determine any differences in pre- and post-scores between the three groups. Statistical significance was set at *p* = 0.05. A multivariate linear regression analysis was conducted to determine if emotional safety impacted development of semantic memory for all groups. A second regression analysis was performed to identify if emotional safety impacted development of procedural memory for the students enrolled within courses with direct equine interaction (Group B).

## 3. Results

### 3.1. Two-Part Self-Reporting Survey Instrument

Emotional Safety Self-Evaluation. For the pre-evaluation for the emotional safety self-reporting survey instrument, the number of participants for Groups A, B, and C were 62, 63, and 46, respectively, with response rates being 44% (Group A), 90% (Group B), and 65% (Group C). In the post-evaluation, the number of participants within each group was 57 (Group A), 63 (Group B), and 45 (Group C), respectively, with response rates at 40%, 90%, and 64%, respectively. There was a 100% response rate when the pre- and post-respondents were matched. Despite lowering in total scores closer to the “ideal” range of 60 that was observed in both equine courses, there were no significant differences in any category of emotional safety (personal security, respect, self-esteem, connectivity) nor in the total score for emotional safety for any of the three groups when comparing pre- and post-evaluations (*p* > 0.05; Table 7).

Semantic Memory: Knowledge Evaluation. Participation numbers and response rates for both pre- and post-evaluations were the same for the knowledge evaluation as seen in the self-reporting emotional safety survey instrument. A significant decrease between pre- and post-knowledge evaluation scores was found in the equine care category for Group A (*p* < 0.05; Table 8). While both groups with equine courses demonstrated significant increases, Group B demonstrated this increase in the grooming/tacking category (*p* < 0.05) and Group C was in the equine behavior category (*p* < 0.05). Although Group A was the only group that dropped in total scores, none of the groups demonstrated a significant difference between pre- and post-evaluations for total scores (*p* > 0.05). Although Group A (R = 0.00, R^2^ = 0.00) showed no predictive value between emotional safety and semantic memory, the regression analysis showed a weak negative correlation between scores for emotional safety and the knowledge evaluation for Group B (R = −0.55, R^2^ = 0.28) and Group C (R = −0.25, R^2^ = 0.06), indicating that as emotional safety scores moved lower to the “ideal” score of 60 that knowledge scores rose.

### 3.2. Procedural Memory: Skill Evaluation

For the skill evaluation, 69 out of the 70 students available from the courses with direct equine interaction (Group B) participated in the pre- and post-evaluations for assessing procedural memory development *(n* = 69). Significant improvements in procedural memory were found in the following categories for the evaluation: Abilities in Barn Management, Quality of Barn Management, Abilities in Equine Handling, and Skills in Team Building (*p* < 0.05; Table 9). However, no significant difference was found in the total score for the skill evaluation (*p* > 0.05). The regression analysis lacked a correlation between scores for emotional safety and the skill evaluation for Group B (R = −0.15, R^2^ = 0.02), and when skill and knowledge scores were combined for Group B, a predictive relationship with emotional safety was still lacking for Group B (R = −0.17, R^2^ = 0.03).

## 4. Discussion

Effective teaching requires the instructor to create an environment that is conducive to learning [4]. Further, with the evolution of the educational environment within the past few years leading towards a trend to online classroom formatting and less hands-on learning, determining the most effective way to approach teaching in today’s classroom will assist students as they make this transition into a nontraditional classroom environment for e-learning [5]. Although animal interaction has been documented to be useful within the educational environment for supporting an environment conducive to learning [7,26,38], research specific to equine interaction, whether it is direct or indirect, within the agricultural-based college classroom has been limited as to whether the horse might be a valuable tool in creating an emotionally safe environment for learning. With the future of educational environments moving more towards e-learning [2,3,6], understanding the value of equine interaction and the effectiveness of indirect interaction can be useful in moving forward with curriculum development within the agricultural-based classroom.

### 4.1. Emotional Safety

Horses were selected for the animal interaction portion of this study due to previous work indicating the value of the animal in promoting components of emotional safety in students [8,38,39,40]. At the end of the semester, total scores for emotional safety, whether students directly or indirectly interacted with the horse, demonstrated a lack of significant improvement similar to students not enrolled in courses with equine interaction. It is, however, important to note that, although insignificant, the course with indirect access to the horse was the only group that saw a decrease in scores in all categories of emotional safety including total scores. Due to initial scores being well over the 60-point “ideal” score for emotional safety, this decrease in emotional safety scores within all categories of emotional safety indicates a potential development of an emotionally safe learning environment; thus, this trend holds promise for the potential of creating an educational environment conducive for learning through the indirect interaction of the horse. This is critical with the potential of virtual learning being a part of future course formatting within the educational environment [2,3,4,6]. In addition, the costs and labor associated with providing students with live interaction with the horse can be daunting for educators, and so, indirect interaction may be a viable option [21]. Interestingly, this lowering to the “ideal” emotional safety score in all categories was not observed within the group with direct equine interaction as personal security scores rose, although insignificant. This may be a reflection of the distractive nature of the animal within the learning environment that can create frustration for some students due to a subconscious fear resulting from a lack of knowledge on how to redirect, as observed by previous studies [7,8,21]. Further, a lack of improvement of personal security may be reflective of the fact that the courses utilized were introductory-based, so these students may be more hesitant within the equine environment than a more experienced student [8]. Nevertheless, as agricultural-based educators move forward within online learning, these findings hold promise and suggest future research should look into the most effective approach to indirect equine interaction to determine best practices for creating an emotionally safe learning environment for a virtual platform.

### 4.2. Semantic Memory

As for semantic memory development, both groups with equine interaction demonstrated retention of semantic memory within at least one category of the equine knowledge examination. As an instructor, this development of semantic memory is important as both courses were introductory-based equine courses, and with none of the students taking additional equine courses at the time of survey participation, these courses would lay the foundation for future equine courses. As for more specific course content, in the area of equine evaluation, for example, Wulff-Risner and Stewart [21] determined that indirect interaction was a more effective teaching approach for adolescents. This may suggest when moving past introductory-based courses, the selection of whether to use indirect or direct interaction may be more specific to the focus of the course. For memory associated with equine behavior, it was the indirect interaction with the horse that assisted with this aspect of learning. Observation of an animal, even without direct interaction, has proven to be a valuable tool in the general understanding of behavior [27,41,42]. For both groups, nonetheless, as students acquired semantic memory through their equine coursework, a relationship was seen with emotional safety; however, this was a weak correlation limiting the conclusions that can be made at this time between emotional safety and semantic memory. Nonetheless, cognitive information is often acquired through specific events evoking episodic memory, which gives a more personal context to the development of memory [43]. Similarly, Silva dos Santos et al. [7] reported an emotional response to learning even with indirect interaction with animals, finding personal experience gives context to the subject matter being learned. In the end, although the weak correlation observed within this study limits conclusions and emphasizes the importance of further research, these findings hold promise that equine interaction, whether direct or indirect, can potentially promote learning in the form of semantic memory development with the facilitation of an emotionally safe learning environment.

### 4.3. Procedural Memory

Direct interaction with an animal allows for learning by doing [7], whereas indirect interaction may limit this opportunity for kinesthetic learning. For those students that learn more through kinesthetic learning, direct interaction can be a valuable teaching tool, although this form of teaching can be costly and labor-intensive when it comes to equines [21]. Labor demands have been reported by Evans et al. [38] as a potential limitation to offering of equine interaction within the collegiate environment. Similarly, as indicated by Holtcamp et al. [44], the application of hands-on equine interactive activities may be hindered by the costs associated with such programs. As such, costs and labor demands associated with equine interaction within the collegiate setting need to be weighed as to the benefits these interactive activities provide for the student. As for the benefits observed within the current study, instructors reported development of procedural memory in all four categories as observed within the instructor-based evaluation of the students’ equine-based skills. While total scores did not show significant improvement, it is important to note that all students that participated in the direct interaction safely performed all activities throughout the semester with no incidence of injury, both for the students and the horses, as reported by the instructors of these courses. This is further evidence of procedural memory development associated with the skills needed for safely handling the horses during the weekly laboratory activities. Nonetheless, procedural memory development did not correlate with emotional safety, but this relationship may not be established with interaction limited to only once a week. More frequent interaction may need to occur to build such a relationship within a non-therapeutic equine environment [44], particularly within an introductory-based educational environment [39,45]. Curriculum content should be further explored to determine whether procedural memory targeted on specific skills will result in creating emotional safety within the student. Simple skills such as grooming have shown positive physiological and emotional responses in participants [25], but further work is warranted to determine the impact on and the relationship with memory development of the college student.

### 4.4. Limitations of the Study

Although this study has assisted in the understanding of the value of equine interaction within the educational environment of the agricultural-based college classroom, with any survey study, there are limitations. With the survey being voluntary and only offered for two semesters, the number of participants was limited, and response rates could have been improved particularly for the group enrolled in courses with no equine interaction. While the courses utilized for participation did not have a major requirement nor required any prerequisites, the courses were based within an agricultural college and were junior and senior level courses, and thus, expanding recruitment outside of these areas may have improved participation numbers. Expansion, in particular, may also allow for investigation of courses outside of those focused on introductory-based activities. A more intensive curriculum beyond just basic handling activities within those with direct interaction with the horse may have more of an impact on the emotional safety of the students [44], however, as observed by Baldwin et al. [25] the simple activity of grooming demonstrated significant emotional and physiological responses within participants of equine interactive activities. Further, while the use of both paper and digital formats for the survey assisted in promoting participation, allowing for format selection based off of convenience and preference, the length of the survey may have been a deterrent to participation [46]. With further testing of the survey instrument, questions can be streamlined to reduce survey length and the associated time commitment from study participants.

Another limitation to this study may be the resistance of students to verbalize aspects of their mental health. The emotional safety evaluation in a survey format may be difficult for students to openly express their feelings due to the negative stigma associated with mental health challenges [12,47,48,49]. In a previous study concerning at-risk youth, researchers implemented the use of oral surveying methods through both group and individual interviews with the researchers [8]. Researchers were able to take that in-person opportunity to question an area of emotional safety more intensely during the interviews by responding to the interviewee’s body language and even asking for specific incidences supporting improvement in emotional safety. Further, the use of the in-person oral surveying methodology may reduce potential subjective, biased responses from the students as the interviewer can further question specific responses based off of body language [8]. However, more objective measures, including physiological responses, may assist in future studies to avoid potential self-reporting bias [38]. An additional benefit to the in-person oral surveying methodology is the improved response rate finding; unlike the current study, the use of the in-person oral surveying methodology resulted in a 100% response rate before and after the research period, according to Cagle-Holtcamp et al. [8].

Finally, while components of the skill evaluation showed significant improvements, the total scores did not increase. This could be attributed to the use of different instructors serving as evaluators due to the lengthy process and limitations of a single instructor’s availability [50]. Other equine-based survey studies have utilized self-reporting surveys, instead of instructor skill evaluations, so that the students can assess their skill level, and thus, reducing instructor variability and the instructor’s time commitment to the evaluations [19,51]. While a self-reporting survey would have allowed all groups within this study to safely participate in this assessment, this type of evaluation can promote personal biases and may not reflect actual skills. Further testing of whether student self-reporting skill evaluations reflect similar results as instructor evaluations may be valuable in minimizing the time and labor involved with this type of assessment of procedural memory development. Nonetheless, the use of self-reporting can also reflect the student’s overall confidence, even if the confidence is based on the student’s perceived equine-based skills [38,39,45], and that observation of student confidence can be of value in further evaluating the areas of emotional safety.

## 5. Conclusions

While students might enjoy the interaction of the live horse within their course activities, the labor and costs associated with direct equine interaction may not be warranted unless the educator is looking to achieve procedural memory development in the form of safe equine handling skills performed by the student or semantic memory development specific to equine grooming and tacking. Furthermore, the relationship between emotional safety and semantic memory observed in both indirect and direct equine interaction suggests the opportunity for effective teaching without the additional burden of utilizing a live animal in the educational environment. As such, with academia moving forward into a more regularly utilized e-learning format, investigating the impact of indirect animal interaction within the collegiate setting may hold promise for building an emotionally safe environment for learning within the online agricultural-based classroom.

## Figures and Tables

**Table 1:** Questions associated with the personal security portion of the emotional safety evaluation that was completed by each college student.

Question Number	Question
1	In the last month, how often have you felt secure in your daily life?
2	In the last month, how often have you felt that you have received enough attention?
3	In the last month, how often have you felt in control of your life?
4	In the last month, how often have you felt a strong connection with friends?
5	In the last month, how often have you had the time for reflection?
6	In the last month, how often have you interacted with people from your local community?
7	In the last month, how often have you engaged in hobby/sport activities with others?
8	In the last month, how often have you felt valued and respected by your friends?
9	In the last month, how often have you felt that there are people who need you?
10	In the last month, how often have you felt that life is meaningful?
11	Over the last two weeks, I felt nervous, anxious, or on edge
12	Over the last two weeks, I was not able to stop or control worrying
13	Over the last two weeks, I worried too much about different things
14	Over the last two weeks, I had trouble relaxing
15	Over the last two weeks, I was so restless that it was hard to sit still
16	Over the last two weeks, I became easily annoyed or irritable
17	Over the last two weeks, I felt afraid as if something awful might happen.

**Table 2:** Questions associated with the respect portion of the emotional safety evaluation that was completed by each college student.

Question Number	Question
18	I am loyal to people who are not present.
19	I gossip or make discounting statements about others.
20	I exaggerate or over dramatize a situation.
21	I make remarks, and use humor or language inappropriately.
22	I admit mistakes; say I am sorry, apologize
23	I give people my undivided attention.
24	My behavior matches what I say I value
25	I treat conversations with respect.
26	I follow through with commitments, conversations, and people.
27	I do the right thing even though it is not fair.
28	I make and keep specific commitments.
29	I communicate when I cannot keep a commitment.
30	I am on time.
31	I am accessible.
32	I have an effective response to problems.
33	I have integrity.
34	I am organized.

**Table 3:** Questions associated with the self-esteem portion of the emotional safety evaluation that was completed by each college student.

Question Number	Question
35	I feel that I’m a person of worth, at least on an equal par with others.
36	I feel that I have a number of good qualities.
37	All in all, I am inclined to feel that I’m a failure.
38	I am able to do things as well as most other people.
39	I feel I do not have much to be proud of.
40	I take a positive attitude toward myself.
41	On the whole, I am satisfied with myself.
42	I wish I could have more respect for myself.
43	I certainly feel useless at times.
44	At times I think that I am no good at all.
45	I can honestly say that I love myself.
46	When I look at myself in the mirror, I am happy with what I see.

**Table 4:** Questions associated with the connectivity portion of the emotional safety evaluation that was completed by each college student.

Question Number	Question
47	I feel disconnected from the world around me.
48	Even around people I know, I do not feel that I really belong.
49	I feel so distant from people.
50	I have no sense of togetherness with my peers.
51	I do not feel related to anyone.
52	I catch myself losing all sense of connectedness with society.
53	I do not feel that I participate with anyone or any group.
54	I feel more comfortable when someone is constantly with me.
55	I’m more at ease doing things with others.
56	Working side by side with others is more comfortable than working alone.
57	It is hard for me to use my skills and talents without someone beside me.
58	I stick to my friend group.
59	I join groups more for the friendship than the activity.
60	I wish to find someone who can be with me all the time.

**Table 5:** Questions used in the equine knowledge exam for the knowledge evaluation that was completed by each college student.

Question Number	Question
1	The first choice of a frightened horse is to:
2	When approaching a horse, you should approach:
3	A horse should be tied up with:
4	When brushing a horse, you should:
5	When riding, your eyes should be:
6	To stop your horse, you should:
7	When riding at the trot, you should keep your hands:
8	When approaching a horse in a stall, what should you do first:
9	When mounting your horse, you should never:
10	When leading a horse safely, you should:
11	Water buckets should be replenished:
12	Stalls are cleaned to:
13	Feeding your horse can get your fingers bit.
14	When leading with a lead rope, you can wrap the excess rope around your hand.
15	You should speak to your horse before touching him.
16	Saddling is done from the left side.
17	When riding, your heels should be higher than your toes.
18	On trail rides, you should not let. Your horse eat grass.
19	Going uphill, lean back for better comfort and safety.
20	Clothes and shoes you wear for riding do not matter except if you are showing.
21	A rubber currycomb is used to scrub the horse’s hair coat in a circular manner.
22	Squeezing with your heels and leaning forward tells your horse to stop.

**Table 6:** Questions used in the skill evaluation that the instructor completed for each college student participating in direct interaction with the horse (Group B).

Question Number	Question
1	Confident when entering horse stall, paddock, and/or pasture and while approaching horse.
2	Able to pick stall and/or clean around horse area (grooming/tacking area) properly and thoroughly.
3	Able to carry and handle properly horse equipment/items (water buckets, tack, etc).
4	Works diligently to complete intensive barn duty (cleans facility and equipment thoroughly, puts attention on the details, works until job is complete).
5	Able to follow directions and complete horse-related tasks to the desired standard.
6	Confident when leading a horse.
7	Confident when grooming a horse.
8	Confident when tacking a horse.
9	Engaged in hands on activities (interacts with others during activity, responds appropriately, etc).
10	Engaged in visual demonstrations (patient, observant, utilized information from demonstration, etc).

**Table 7:** Means (standard deviations) of the emotional safety self-evaluation at the beginning (pre) and end (post) of the semester for Groups A, B, and C. Paired *t*-tests performed for pre- and post-scores within each group with *p*-values given (*p* = 0.05).

		Group A	Group B	Group C
Personal Security	Pre	36.07 (8.94)	35.06 (10.41)	35.96 (8.06)
	Post	35.84 (10.42)	35.58 (8.93)	34.80 (11.06)
	*p*-value	0.88	0.77	0.58
Respect	Pre	27.21 (4.96)	26.74 (6.89)	28.63 (6.67)
	Post	27.26 (7.40)	25.68 (6.94)	27.39 (8.30)
	*p*-value	0.96	0.39	0.39
Self-Esteem	Pre	22.50 (6.68)	22.89 (8.29)	22.65 (6.58)
	Post	22.90 (8.11)	21.91 (8.31)	21.85 (8.35)
	*p*-value	0.72	0.51	0.60
Connectivity	Pre	30.05 (7.27)	31.45 (8.44)	29.91 (6.56)
	Post	31.00 (7.84)	28.87 (7.23)	28.35 (9.07)
	*p*-value	0.45	0.08	0.36
Total Scores	Pre	115.83 (22.59)	116.13 (29.81)	117.15 (23.77)
	Post	117.00 (29.62)	112.04 (26.37)	112.39 (33.28)
	*p*-value	0.78	0.42	0.44

Groups were categorized according to the following: Group A—students enrolled in courses with no equine interaction; Group B—students enrolled in courses with direct equine interaction; and Group C—students enrolled in courses with indirect equine interaction.

**Table 8:** Means (standard deviations) of the knowledge evaluation at the beginning (pre) and end (post) of the semester for Groups A, B, and C. Paired *t*-tests performed for pre- and post-scores within each group with *p*-values given (*p* = 0.05).

		Group A	Group B	Group C
Grooming/Tacking	Pre	4.28 (1.06)	4.28 (0.84)	4.63 (0.74)
	Post	4.34 (0.97)	4.57 (0.67)	4.61 (0.68)
	*p*-value	0.70	0.03	0.89
Riding Knowledge	Pre	6.95 (1.08)	7.23 (1.12)	7.00 (1.32)
	Post	6.64 (1.65)	7.17 (1.28)	7.24 (1.06)
	*p*-value	0.18	0.77	0.19
Equine Behavior	Pre	3.45 (0.63)	3.57 (0.67)	3.59 (0.78)
	Post	3.22 (0.82)	3.66 (0.68)	3.80 (0.40)
	*p*-value	0.06	0.35	0.04
Equine Care	Pre	4.69 (0.57)	4.49 (0.78)	4.54 (0.66)
	Post	4.33 (1.02)	4.53 (0.70)	4.54 (0.66)
	*p*-value	0.00	0.70	1.00
Total Scores	Pre	19.36 (2.26)	19.57 (2.41)	19.76 (2.68)
	Post	18.53 (3.57)	19.92 (2.20)	20.20 (1.75)
	*p*-value	0.08	0.31	0.24

Groups were categorized according to the following: Group A—students enrolled in courses with no equine interaction; Group B—students enrolled in courses with direct equine interaction; and Group C—students enrolled in courses with indirect equine interaction.

**Table 9:** Means (standard deviations) of the skill evaluation scores at the beginning (pre) and end (post) of the semester for students enrolled in courses with direct equine interaction (Group B). Paired *t*-tests performed for pre- and post-scores with *p*-values given (*p* = 0.05).

		Scores
Barn Management	Pre	8.13 (11.09)
	Post	9.84 (1.83)
	*p*-value	0.00
Quality in Barn Management	Pre	5.43 (1.35)
	Post	6.58 (1.23)
	*p*-value	0.00
Abilities in Equine Handling	Pre	7.14 (2.78)
	Post	9.45 (1.94)
	*p*-value	0.00
Skills in Team Building	Pre	5.51 (1.26)
	Post	6.67 (1.17)
	*p*-value	0.00
Total Scores	Pre	32.54 (5.50)
	Post	36.09 (14.68)
	*p*-value	0.08

## Data Availability

The data presented in this study are available upon request from the corresponding author. The data are not publicly available to ensure confidentiality of study participants.
